# Synthesis of *N*-(3-Acyloxyacyl)glycines, Small Molecules with Potential Role in Gut Microbiome-Endocannabinoidome Communication

**DOI:** 10.3390/molecules29153703

**Published:** 2024-08-05

**Authors:** Rosaria Villano, Vincenzo Di Marzo

**Affiliations:** 1 Istituto di Chimica Biomolecolare, CNR, Via Campi Flegrei 34, 80078 Pozzuoli, NA, Italy; vincenzo.di-marzo.1@ulaval.ca; 2 Canada Excellence Research Chair on the Gut Microbiome-Endocannabinoidome Axis in Metabolic Health, Faculty of Medicine and Faculty of Agricultural and Food Sciences, Centre de Recherche de l’Institut de Cardiologie et Pneumologie de l’Université et Institut sur la Nutrition et les Aliments Fonctionnels, Centre NUTRISS, Université Laval, Quebec City, QC G1V 4G5, Canada

**Keywords:** organic synthesis, *N*-(3-acyloxyacyl)glycines, *N*-acyl amino acids, gut microbiota metabolites, endocannabinoidome

## Abstract

The synthesis of some *N*-(3-acyloxyacyl)glycines, an interesting class of bioactive gut microbiota metabolites, is described. This procedure involves seven reaction steps using the commercially available Meldrum’s acid to obtain highly pure products, in normal or deuterated form. The key point of the synthetic strategy was the use of commendamide *t*-butyl ester as a synthetic intermediate, a choice that allowed the removal of the protecting group at the end of the synthetic procedure without degrading of the other ester bond present in the molecule. The developed synthetic sequence is particularly simple, uses readily available reagents and involves a limited number of purifications by chromatographic column, with a reduction in the volume of solvent and energy used.

## 1. Introduction

**Figure 1:**
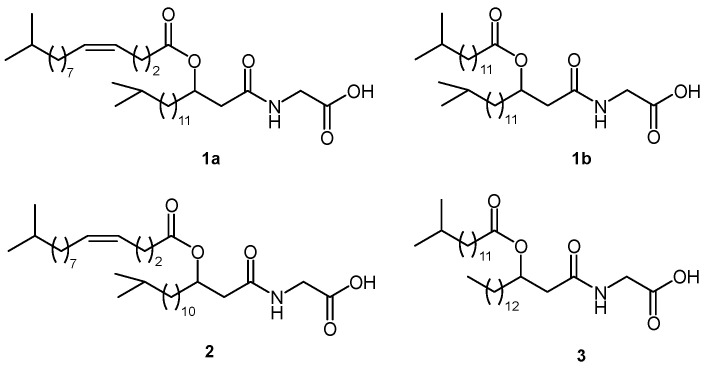
Some bioactive *N*-(3-acyloxyacyl)glycines reported in the literature.

*N*-(3-acyloxyacyl)glycines are an important class of bioactive products of bacterial origin. Molecules of this type were isolated by Morishita et al. from a marine bacterium, *Cytophaga* sp. SANK 71996, [1] and, through a refined work of structural elucidation, it was possible to identify them. The most active molecules among those isolated had branched-chain acyl groups (**1a**, **1b** and **2**, Figure 1) and proved to be highly selective inhibitors for N-type calcium channels (localized primarily in the nervous system and involved in the release of neurotransmitters) vs. L-type channels.

Natural products with a similar structure, known as Topostins B, have also been isolated from a culture broth of *Flexibacter topostinus* sp. Nov. [2] and in this case, after the structural identification, their bioactivity as inhibitors of mammalian DNA topoisomerase I was also highlighted (products **1b** and **3**, Figure 1) [3]. It should also be noted that some of these molecules (for example Topostin B567) have the same structure as the metabolites produced by the marine bacterium *Cytophaga* sp. SANK 71996 (in this case, **1b**). Interestingly, *N*-(3-acyloxyacyl)glycines bear a chemical resemblance to mammalian linear fatty acid esters of hydroxyl fatty acids (FAHFAs), which also contain an ester bond between the two chains (described as the “estolide” bond), although it is from the C-5 of the lower chain, and possess key biological activities in the regulation of diabetes and inflammation [4].

Against this background, the recent discovery that even bacteria belonging to the mammalian gut microbiome (*Bacteroides thetaiotaomicron*) are able to produce *N*-(3-acyloxyacyl)glycines appears particularly noteworthy [5,6]. In particular, Lynch et al. identified a gene from *Bacteroides*, *glsB* (BT_3459), which encodes an *N*-acyltransferase required for the initial production of *N*-(3-hydroxyacyl)glycines such as commendamide [7,8] (a gut microbiota-derived bioactive metabolite with a very similar structure to long-chain *N*-acyl amino acids belonging to the endocannabinoidome [9]), and another immediately close gene, *glsA* (BT_3458), which encodes an *O*-acyltransferase, required for the subsequent esterification of the hydroxy group with endogenous or dietary fatty acids.

Differently from what has been reported for other types of bacteria, the biosynthesis of *N*-(3-acyloxyacyl)glycines with linear acyl chains (such as *N*-[3-(palmitoyloxy)palmitoyl]glycine, i.e., the derivative of commendamide acylated with palmitic acid [5]), seems possible in the case of the mammalian gut microbiota and this different situation might be explained considering that, in this case, linear long-chain fatty acids are widely available in the host (as endogenous or dietary precursors), and, therefore, they can be used as synthons for the biosynthesis of *N*-(3-acyloxyacyl)glycines.

Some of these *N*-(3-acyloxyacyl)glycines seem to play a fundamental role in the adaptation processes of *Bacteroides* to environmental stress but also in the colonization process of the mammalian gut by beneficial bacteria. The identities of most of these molecules were inferred by tandem MS/MS analysis although, by using only this technique, sometimes it was not possible to demonstrate the presence of straight- or branched-chain acyl groups in the molecules with certainty [5].

The discovery that gut microbiota are able to biosynthesize *N*-(3-acyloxyacyl)glycines is also extremely important because, in this case, potentially bioactive molecules are immediately bioavailable in the host, considering that gut microbiota live in symbiosis with the latter and therefore these metabolites could have a direct impact on human physiology. In this regard, it is important to note that varied experimental evidence highlights the close interaction between the gut microbiome and the endocannabinoidome, with microbial metabolites that can interact with molecular targets of the endocannabinoidome and vice versa (the gut microbiome–endocannabinoidome axis), [10] which is also thanks to the structural similarities that often exist between microbial metabolites and endogenous signal molecules, as highlighted above. All this considered, the identification of new molecules that can act as chemical signals in host–microbe communication, but also the understanding of the molecular mechanisms underlying this communication, is fundamental and, in this context, the *N*-(3-acyloxyacyl)glycines seem to be able to play an important role.

Organic synthesis [11] is fundamental for the chemical and biomolecular study of the microbiome, not only for the production of greater amounts of natural products with the aim of extending their pharmacological screening and identifying new molecular mechanisms of interaction with the host, but also to achieve unequivocal structural identification of new metabolites (thanks to the synthesis of pure standards that can be compared to products extracted from biological matrices). Therefore, the elaboration of simple and effective synthetic protocols that, if necessary, can also be used for the synthesis of products in deuterated form (useful in drug discovery [12] and for the development of LC-MS quantitative analysis methods), is very important. In fact, in the development of new LC-MS methods for the quantification of analytes in biological matrices, the use of internal standards [13,14] is fundamental and, in this context, a stable isotope-labeled form of the molecule to be quantified (such as the deuterated analogue, which is not prone to exchange/loss of the isotopic label) is the best internal standard, because it has chemical–physical properties very similar to the analyte (the same extraction recovery, ionization response in ESI-MS, and chromatographic retention time) so it can be co-eluted with the analyte. However, their molecular masses are well distinguishable. On the other hand, the structural properties of *N*-(3-acyloxyacyl)glycines and the bioactivities highlighted for many molecules belonging to this class, as well as the presence of a scaffold found also in other bioactive products (WB-3559 A, B, C and D; Flavolipin; FAHFAs; etc.), [4,15,16] make them an interesting target for chemical synthesis.

In this work, an easy and versatile methodology for the synthesis of some *N*-(3-acyloxyacyl)glycines was developed, starting from the synthetic strategy previously reported for commendamide and its analogues [17]. We describe a synthetic sequence characterized by the use of simple workups, readily available reagents and minimal volumes of organic solvents.

## 2. Results and Discussion

**Scheme 1:**
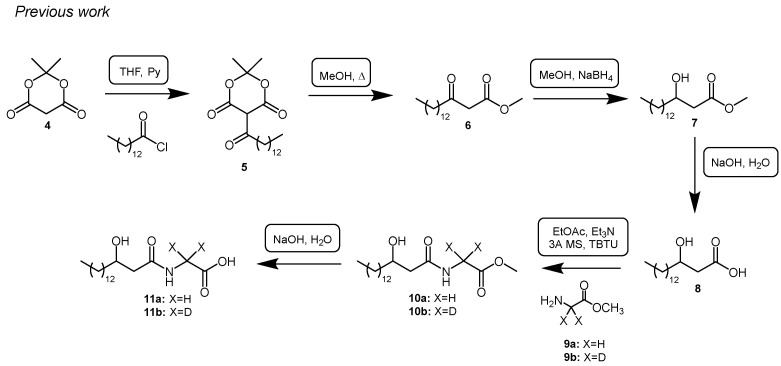
Synthesis of normal and deuterated commendamide **11** [17].

The synthesis of commendamide and its analogues was recently carried out in six steps, starting with the acylation of Meldrum’s acid **4** with a suitable acyl chloride (Scheme 1) [17]. Interestingly, none of the steps involved the use of halogenated solvents and most of the reactions proceeded with high yields, so it was often possible to work directly on the unpurified reaction crudes. In fact, in the multi-step synthesis reported in Scheme 1, only two chromatographic column purifications are required with considerable simplification of the experimental procedures and reduction in the volume of solvent and energy used.

With the aim of synthesizing some *N*-(3-acyloxyacyl)glycines between commendamide and bioavailable long-chain fatty acids (endogenous or dietary LCFA), the synthetic strategy reported in Scheme 1 was applied to obtain the intermediates **10**; then, the products **10a** and **10b** were submitted to an esterification reaction with oleoyl chloride **12** before the subsequent basic hydrolysis.

**Scheme 2:**
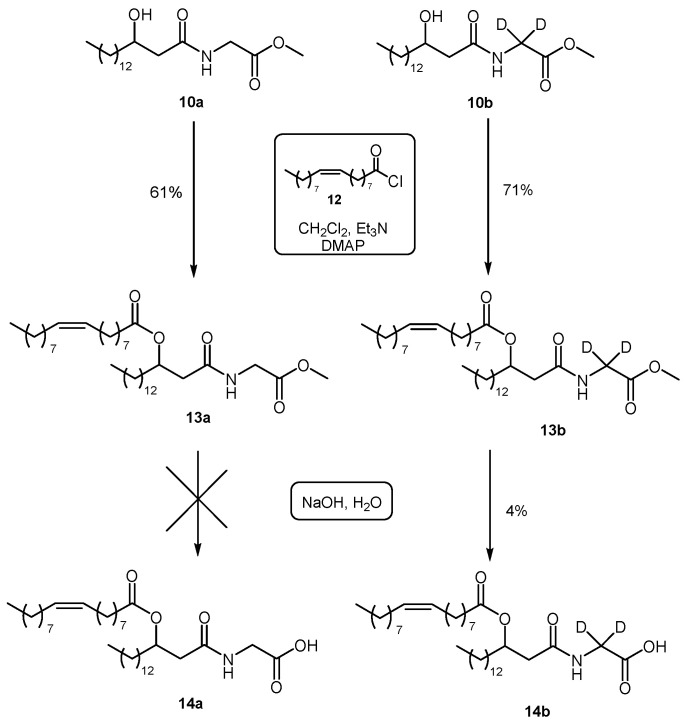
Attempted sequence for the synthesis of *N*-(3-acyloxyacyl)glycines **14**, starting from the intermediates **10**.

**Scheme 3:**
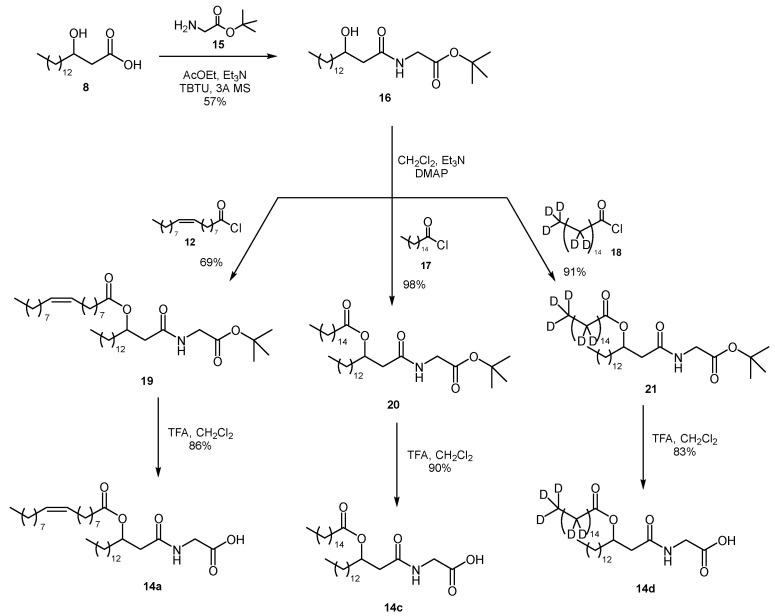
Modified synthetic sequence for the production of *N*-(3-acyloxyacyl)glycines **14**.

Unfortunately, basic hydrolysis led to the complete degradation of the intermediate **13a** (also at low temperature), with consequent formation of commendamide **11a** and oleic acid, due to the hydrolysis of both ester bonds present in the molecule. Therefore, it was not possible to obtain the product **14a**, while the deuterated product **14b** was isolated only in a very small amount (Scheme 2). An alternative method for the selective hydrolysis of methyl esters with LiCl-DMF [18] was ineffective on the substrates **13**. Thus, a modified synthetic sequence was used in order to obtain the products **14**. Indeed, 3-hydroxypalmitic acid **8**, synthesized as shown in Scheme 1, was subjected to a coupling reaction with glycine *t*-butyl ester **15** (Scheme 3), instead of glycine methyl ester **9**, as reported also by Morishita et al. for the synthesis of similar products [1].

The corresponding commendamide *t*-butyl ester **16** was then esterified with three different long-chain fatty acid chlorides (oleoyl-chloride **12**, palmitoyl-chloride **17** and its deuterated analogue **18**) with good yields, and the obtained products (**19**, **20** and **21**) were finally deprotected by treatment with trifluoroacetic acid (TFA) at room temperature to yield *N*-(3-acyloxyacyl)glycines **14**. In fact, TFA was able to selectively remove *t*-butyl esters at rt, without hydrolyzing the other ester bond present in the molecule. Thanks to this modified procedure (Scheme 3), the products **14** were obtained with a total yield of up to 40% starting from Meldrum’s acid **4** (seven steps).

## 3. Materials and Methods

### 3.1. General

All reagents and solvents were Merck-SigmaAldrich and used as received. The reactions were monitored by thin-layer chromatography (TLC) on Merck silica gel plates (0.25 mm) and visualized by UV light at 254 nm and cerium sulfate reagent. ^1^H NMR and ^13^C NMR spectra were recorded on a Bruker DRX 600 spectrometer equipped with a three-channel inverse TCI CryoProbe (Bruker, Milan, Italy) at room temperature in CDCl_3_. Chemical shifts are reported in ppm; multiplicities are indicated by s (singlet), d (doublet), t (triplet), q (quartet), m (multiplet), and br (broad). Coupling constants (J) are reported in Hz. IR measurements were conducted on KBr pills with Vertex 70 Bruker spectrophotometer (Bruker, Milan, Italy) and maximum absorptions are reported in wavelength (cm^−1^). High-resolution mass spectra (HRMS) were acquired using an OrbiTrap high-resolution mass spectrometer (Q Exactive) equipped with the heated electrospray ionization probe HESI-II (Thermo Fisher Scientific, Bremen, Germany) operating in both positive and negative ion modes. Yields are given for isolated products showing one spot on a TLC plate and no impurities detectable in the NMR spectrum. The synthetic procedures and spectroscopic characterizations of products **6**, **7**, **8**, **10a** and **10b** are reported in the literature [17]. ^1^H and ^13^C NMR spectra of products **13a**, **13b**, **16**, **19**, **20**, **21**, **14a**, **14b**, **14c** and **14d** are reported in Supplementary Materials.

### 3.2. Procedures

Methyl [3-((9*Z*)-octadec-9-enoyloxy) hexadecanoyl]glycinate or methyl [3-(oleoyloxy)palmitoyl]glycinate (**13a**). A solution of the product **10a** (0.12 mmol) in CH_2_Cl_2_ (4.0 mL) was prepared in a vial. The reaction was cooled at 0 °C and, in the presence of a catalytic amount of DMAP, Et_3_N (2.3 eq, 0.28 mmol) was added; then, oleoyl chloride (2.3 eq, 0.28 mmol) was also added slowly. The reaction mixture was stirred at 0 °C for 15 min and then for 24 h at rt. After adding H_2_O (4 mL), the phases were separated, and the H_2_O phase was extracted with CH_2_Cl_2_ (2 × 3 mL). The combined organic layers were dried over Na_2_SO_4_ and the solvent was removed by reduced pressure; the crude product was purified by silica gel chromatography (light petroleum ether/EtOAc 9/1 to 7/3) to give the product **13a** (44.3 mg, 0.073 mmol, 61% yield, pale yellow oil).

R_f_: 0.4 (silica gel, petroleum ether/EtOAc = 7:3).

IR (KBr): 3327, 2958, 1735, 1675, 1467, 1200 cm^–1^.

^1^H NMR (600 MHz, CDCl_3_): δ = 6.26 (bs, 1H), 5.34 (m, 2H), 5.16 (m, 1H), 4.03 (d, J = 5.1 Hz, 2H), 3.75 (s, 3H), 2.54 (dd, J = 14.7, 6.8 Hz, 1H), 2.49 (dd, J = 14.7, 5.34 Hz, 1H), 2.31 (t, J = 7.6 Hz, 2H), 2.01 (m, 4H), 1.69–1.58 (m, 4H), 1.35–1.24 (m, 42H), 0.88 (t, J = 6.8 Hz, 6H).

^13^C NMR (150 MHz, CDCl_3_): δ = 173.5, 170.3, 169.9, 130.0, 129.7, 71.1, 52.4, 41.4, 41.2, 34.5, 34.1, 31.93, 31.91, 29.8, 29.72, 29.69, 29.68, 29.66, 29.64, 29.56, 29.53, 29.5, 29.4, 29.3 (3C), 29.2, 29.14, 29.1, 27.23, 27.18, 25.3, 24.9, 22.7 (2C), 14.1 (2C). 

HRMS (ESI): m/z [M+Na]^+^ calcd for C_37_H_69_NNaO_5_: 630.5068; found: 630.5072.

Methyl [3-((9*Z*)-octadec-9-enoyloxy) hexadecanoyl]glycinate-d_2_ or methyl [3-(oleoyloxy)palmitoyl]glycinate-d_2_ (**13b**). A solution of the product **10b** (0.15 mmol) in CH_2_Cl_2_ (5.0 mL) was prepared in a vial. The reaction was cooled at 0 °C and, in the presence of a catalytic amount of DMAP, Et_3_N (2.3 eq, 0.35 mmol) was added; then, oleoyl chloride (2.3 eq, 0.35 mmol) was also added slowly. The reaction was stirred at 0 °C for 15 min and then for 24 h at rt. After adding H_2_O (5 mL), the phases were separated and the H_2_O phase was extracted with CH_2_Cl_2_ (2 × 5 mL). The combined organic layers were dried over Na_2_SO_4_ and the solvent was removed by reduced pressure; the crude product was purified by silica gel chromatography (light petroleum ether/EtOAc 9/1 to 7/3) to give the product **13b** (67.1 mg, 0.11 mmol, 71% yield, pale yellow oil).

R_f_: 0.4 (silica gel, petroleum ether/EtOAc = 7:3).

IR (KBr): 3319, 2954, 1730, 1656, 1467, 1210 cm^–1^.

^1^H NMR (600 MHz, CDCl_3_): δ = 6.23 (bs, 1H), 5.34 (m, 2H), 5.16 (m, 1H), 3.76 (s, 3H), 2.54 (dd, J = 14.7, 6.8 Hz, 1H), 2.49 (dd, J = 14.7, 5.34 Hz, 1H), 2.31 (t, J = 7.4 Hz, 2H), 2.01 (m, 4H), 1.69–1.58 (m, 4H), 1.35–1.24 (m, 42H), 0.88 (t, J = 6.8 Hz, 6H).

^13^C NMR (150 MHz, CDCl_3_): δ = 173.5, 170.3, 169.9, 130.0, 129.7, 71.1, 52.4, 41.4, 34.5, 34.1, 31.93, 31.91, 29.8, 29.72, 29.69, 29.68, 29.66, 29.64, 29.56, 29.53, 29.5, 29.4, 29.3 (3C), 29.2, 29.1 (2C), 27.23, 27.18, 25.3, 24.9, 22.7 (2C), 14.1 (2C).

HRMS (ESI): m/z [M+Na]^+^ calcd for C_37_H_67_D_2_NNaO_5_: 632.5193; found: 632.5198.

*N*-[3-((9*Z*)-octadec-9-enoyloxy)hexadecanoyl]glycine-d_2_ or *N*-[3-(oleoyloxy)palmitoyl]glycine-d_2_ (**14b**)**.** To a solution of **13b** (0.09 mmol) in THF (1.0 mL) at 0 °C, 1N NaOH_aq_ (2 eq, 0.18 mmol) was added. The mixture was stirred at 0 °C for 1h and at rt for 30 min, then acidified with 1N HCl. After adding H_2_O (4 mL) and EtOAc (3 mL), the phases were separated and the H_2_O phase was extracted with EtOAc (2 × 4 mL). The combined organic layers were dried over Na_2_SO_4_ and evaporated; the residue was washed with *n*-hexane (2 × 1 mL) and *n*-hexane/EtOAc 9/1 (2 × 1 mL) and dried under vacuum to afford the product **14b** (2.4 mg, 0.004 mmol, 4% yield, colorless amorphous solid).

IR (KBr): 3351, 2919, 1731, 1627, 1468, 1235, 1203 cm^–1^.

^1^H NMR (600 MHz, CDCl_3_): δ = 6.38 (bs, 1H), 5.34 (m, 2H), 5.16 (m, 1H), 2.56 (dd, J = 14.8, 6.8 Hz, 1H), 2.51 (dd, J = 14.8, 5.3 Hz, 1H), 2.30 (t, J = 7.5 Hz, 2H), 2.01 (m, 4H), 1.69–1.58 (m, 4H), 1.35–1.24 (m, 42H), 0.88 (t, J = 6.8 Hz, 6H).

^13^C NMR (150 MHz, CDCl_3_): δ = 173.8, 171.9, 170.6, 130.0, 129.7, 71.1, 41.4, 34.5, 34.4, 34.1, 31.93, 31.91, 29.8, 29.7, 29.69, 29.66, 29.56, 29.53, 29.5, 29.4, 29.3 (3C), 29.2, 29.13, 29.1, 29.0, 27.23, 27.18, 25.2, 24.9, 22.7 (2C), 14.1 (2C).

HRMS (ESI): m/z [M+Na]^+^ calcd for C_36_H_65_D_2_NNaO_5_: 618.5037; found: 618.5041 (in positive ion mode). m/z [M-H]^−^ calcd for C_36_H_64_D_2_NO_5_: 594.5072; found: 594.5068 (in negative ion mode).

*tert*-Butyl (3-hydroxyhexadecanoyl)glycinate (**16**)**.** To a solution of 3-hydroxypalmitic acid **8** (0.65 mmol) in EtOAc (6 mL), Et_3_N (3 eq, 1.95 mmol), MS (1 g) and TBTU (1 eq, 0.65 mmol) were added at rt. After 1 h, glycine *t*-butyl ester **15** (2.5 eq, 1.6 mmol) was added and the reaction mixture was stirred for 6 h at rt. After adding H_2_O (7 mL), the phases were separated and the H_2_O phase was extracted with EtOAc (2 × 7 mL). The combined organic layers were dried over Na_2_SO_4_ and evaporated; the crude product was purified by silica gel chromatography (light petroleum ether/EtOAc 8/2 to 7/3) to give the product **16** (142.6 mg, 0.37 mmol, 57% yield, colorless wax).

R_f_: 0.2 (silica gel, petroleum ether/EtOAc = 7:3).

IR (KBr): 3350, 3291, 2850, 1744, 1719, 1670, 1223 cm^–1^.

^1^H NMR (600 MHz, CDCl_3_): δ = 6.26 (bs, NH, 1H), 4.00–3.89 (m, 3H), 2.42 (dd, J = 15.1, 2.5 Hz, 1H), 2.30 (dd, J = 15.1, 9.2 Hz, 1H), 1.57–1.27 (m, 33H), 0.88 (t, J = 6.8 Hz, 3H).

^13^C NMR (150 MHz, CDCl_3_): δ = 172.6, 169.2, 82.6, 68.7, 42.7, 41.9, 36.9, 31.9, 29.7 (2C), 29.66 (2C), 29.6 (2C), 29.5, 29.4, 28.0 (3C), 25.5, 22.7, 14.1.

HRMS (ESI): *m*/*z* [M+Na]^+^ calcd for C_22_H_43_NNaO_4_: 408.3084; found: 408.3090.

*tert*-Butyl [3-((9*Z*)-octadec-9-enoyloxy)hexadecanoyl]glycinate or *tert*-butyl [3-(oleoyloxy)palmitoyl]glycinate (**19**)**.** A solution of **16** (0.1 mmol) and Et_3_N (2.0 eq, 0.2 mmol) in CH_2_Cl_2_ (3 mL), in the presence of a catalytic amount of DMAP, was prepared; then, also oleoyl chloride (1.7 eq, 0.17 mmol) was added slowly. The reaction was stirred for 2 h at rt then, after adding H_2_O (4 mL), the phases were separated and the H_2_O phase was extracted with CH_2_Cl_2_ (2 × 5 mL). The combined organic layers were dried over Na_2_SO_4_ and the solvent was removed by reduced pressure; the crude product was purified by silica gel chromatography (light petroleum ether/EtOAc 95/5 to 9/1) to give the product **19** (44.8 mg, 0.069 mmol, 69% yield, colorless oil).

R_f_: 0.6 (silica gel, petroleum ether/EtOAc = 7:3).

IR (KBr): 3309, 2954, 1739, 1668, 1460, 1160 cm^–1^.

^1^H NMR (600 MHz, CDCl_3_): δ = 6.20 (bs, 1H), 5.34 (m, 2H), 5.16 (m, 1H), 3.91 (d, J=4.9 Hz, 2H), 2.52 (dd, J = 14.7, 6.8 Hz, 1H), 2.48 (dd, J = 14.7, 5.4 Hz, 1H), 2.30 (t, J = 7.5 Hz, 2H), 2.01 (m, 4H), 1.69–1.58 (m, 4H), 1.47 (s, 9H), 1.35–1.21 (m, 42H), 0.88 (t, J = 6.8 Hz, 6H).

^13^C NMR (150 MHz, CDCl_3_): δ = 173.4, 169.7, 169.0, 130.0, 129.7, 82.3, 71.1, 42.1, 41.4, 34.5, 34.1, 31.93, 31.91, 29.8, 29.7, 29.68 (2C), 29.66 (2C), 29.56, 29.53, 29.5, 29.3 (4C), 29.2, 29.15, 29.14, 28.0 (3C), 27.23, 27.19, 25.2, 25.0, 22.7 (2C), 14.1 (2C).

HRMS (ESI): *m*/*z* [M+Na]^+^ calcd for C_40_H_75_NNaO_5_: 672.5537; found: 672.5541.

*tert*-Butyl [3-(hexadecanoyloxy) hexadecanoyl]glycinate or *tert*-butyl [3-(palmitoyloxy)palmitoyl]glycinate (**20**)**.** According to the synthesis of **19**, **20** (61.1 mg, 0.098 mmol, 98% yield, colorless wax) was prepared starting from **16** (0.1 mmol) and palmitoyl chloride (1.7 eq, 0.17 mmol).

R_f_: 0.6 (silica gel, petroleum ether/EtOAc = 7:3).

IR (KBr): 3300, 2926, 1736, 1675, 1467, 1200 cm^–1^.

^1^H NMR (600 MHz, CDCl_3_): δ = 6.21 (bs, 1H), 5.16 (m, 1H), 3.90 (dd, J = 4.9, 0.7 Hz, 2H), 2.51 (dd, J = 14.7, 6.8 Hz, 1H), 2.47 (dd, J = 14.7, 5.3 Hz, 1H), 2.30 (t, J = 7.5 Hz, 2H), 1.67–1.55 (m, 4H), 1.46 (s, 9H), 1.34–1.21 (m, 46H), 0.87 (t, J = 6.8 Hz, 6H).

^13^C NMR (150 MHz, CDCl_3_): δ = 173.4, 169.7, 169.0, 82.3, 71.1, 42.0, 41.4, 34.5, 34.1, 31.9 (2C), 29.7 (4C), 29.66 (4C), 29.64 (2C), 29.55, 29.51, 29.50, 29.4 (3C), 29.3, 29.2, 28.0 (3C), 25.2, 25.0, 22.7 (2C), 14.1 (2C).

HRMS (ESI): *m*/*z* [M+Na]^+^ calcd for C_38_H_73_NNaO_5_: 646.5381; found: 646.5388.

*tert*-Butyl [3-(hexadecanoyloxy) hexadecanoyl]glycinate-d_31_ or *tert*-butyl [3-(palmitoyloxy)palmitoyl]glycinate-d_31_ (**21**). According to the synthesis of **19**, **21** (43.2 mg, 0.066 mmol, 91% yield, colorless wax) was prepared starting from **16** (0.073 mmol) and deuterated palmitoyl chloride (1.7 eq, 0.12 mmol, prepared from palmitic acid-d_31_ and oxalyl chloride [19]).

R_f_: 0.6 (silica gel, petroleum ether/EtOAc = 7:3).

IR (KBr): 3302, 2941, 1736, 1672, 1467, 1204 cm^–1^.

^1^H NMR (600 MHz, CDCl_3_): δ = 6.20 (bs, 1H), 5.16 (m, 1H), 3.90 (dd, J = 4.9, 0.7 Hz, 2H), 2.51 (dd, J = 14.6, 6.8 Hz, 1H), 2.47 (dd, J = 14.6, 5.4 Hz, 1H), 1.67–1.55 (m, 2H), 1.46 (s, 9H), 1.34–1.21 (m, 22H), 0.87 (t, J = 6.8 Hz, 3H).

^13^C NMR (150 MHz, CDCl_3_): δ = 173.5, 169.7, 169.0, 82.3, 71.1, 42.1, 41.5, 34.1, 31.9, 29.7, 29.68, 29.66 (2C), 29.55, 29.5, 29.4, 29.3, 28.0 (3C), 25.2, 22.7, 14.1.

HRMS (ESI): *m*/*z* [M+Na]^+^ calcd for C_38_H_42_D_31_NNaO_5_: 677.7327; found: 677.7309.

*N*-[3-((9*Z*)-octadec-9-enoyloxy)hexadecanoyl]glycine or *N*-[3-(oleoyloxy)palmitoyl]glycine (**14a**)**.** Trifluoroacetic acid (145.5 µL) was added to a solution of the product **19** (0.030 mmol) in CH_2_Cl_2_ (0.8 mL). After 4 h under stirring at rt, H_2_O (1 mL) was added; the phases were separated and the H_2_O phase was extracted with CH_2_Cl_2_ (2 × 1 mL). The combined organic layers were dried over Na_2_SO_4_ and evaporated; the solid residue was washed with cool *n*-hexane (3 × 0.5mL), recrystallized from n-hexane/CH_2_Cl_2_ and dried under vacuum to afford the product **14a** (15.4 mg, 0.026 mmol, 86% yield, colorless solid).

IR (KBr): 3348, 2924, 1746, 1627, 1467, 1250, 1200 cm^–1^.

^1^H NMR (600 MHz, CDCl_3_): δ = 6.39 (bs, 1H), 5.35 (m, 2H), 5.16 (m, 1H), 4.08 (d, J = 5.2 Hz, 2H), 2.57 (dd, J = 14.8, 6.8 Hz, 1H), 2.53 (dd, J = 14.8, 5.3 Hz, 1H), 2.31 (t, J = 7.5 Hz, 2H), 2.01 (m, 4H), 1.68–1.58 (m, 4H), 1.35–1.21 (m, 42H), 0.87 (t, J = 7.0 Hz, 6H).

^13^C NMR (150 MHz, CDCl_3_): δ = 173.8, 171.8, 170.8, 130.0, 129.7, 71.1, 41.4, 41.3, 34.5, 34.1, 31.93, 31.91, 29.8, 29.7, 29.68 (2C), 29.64, 29.55 (2C), 29.5, 29.4, 29.3 (3C), 29.2, 29.13, 29.11 (2C), 27.23, 27.18, 25.2, 25.0, 22.7, 22.6, 14.1 (2C).

HRMS (ESI): *m*/*z* [M+Na]^+^ calcd for C_36_H_67_NNaO_5_: 616.4911; found: 616.4916 (in positive ion mode). *m*/*z* [M-H]^−^ calcd for C_36_H_66_NO_5_: 592.4946; found: 592.4950 (in negative ion mode).

*N*-[3-(hexadecanoyloxy)hexadecanoyl]glycine or *N*-[3-(palmitoyloxy)palmitoyl]glycine (**14c**)**.** According to the synthesis of **14a**, **14c** (16.4 mg, 0.029 mmol, 90% yield, colorless solid) was prepared starting from **20** (0.032 mmol).

IR (KBr): 3342, 2954, 2891, 1740, 1627, 1468, 1212 cm^–1^.

^1^H NMR (600 MHz, CDCl_3_): δ = 6.45 (m, 1H), 5.16 (m, 1H), 4.05 (d, J = 5.3 Hz, 2H), 2.56 (dd, J = 14.7, 6.9 Hz, 1H), 2.51 (dd, J = 14.7, 5.3 Hz, 1H), 2.30 (t, J = 7.5 Hz, 2H), 1.67–1.55 (m, 4H), 1.34–1.21 (m, 46H), 0.88 (t, J = 6.9 Hz, 6H).

^13^C NMR (150 MHz, CDCl_3_): δ = 173.9, 172.1, 170.7, 71.2, 41.41, 41.39, 34.5, 34.1, 31.9 (2C), 29.7 (4C), 29.69 (2C), 29.67 (4C), 29.57, 29.52 (2C), 29.4 (2C), 29.33, 29.31, 29.2, 25.2, 25.0, 22.7 (2C), 14.1 (2C).

HRMS (ESI): *m*/*z* [M+Na]^+^ calcd for C_34_H_65_NNaO_5_: 590.4755; found: 590.4752 (in positive ion mode). *m*/*z* [M-H]^−^ calcd for C_34_H_64_NO_5_: 566.4790; found: 566.4791 (in negative ion mode).

*N*-[3-(hexadecanoyloxy)hexadecanoyl]glycine-d_31_ or *N*-[3-(palmitoyloxy)palmitoyl]glycine-d_31_ (**14d**)**.** According to the synthesis of **14a**, **14d** (14.9 mg, 0.025 mmol, 83% yield, colorless solid) was prepared starting from **21** (0.030 mmol).

IR (KBr): 3349, 2851, 1740, 1623, 1468, 1202 cm^–1^.

^1^H NMR (600 MHz, CDCl_3_): δ = 6.43 (bs, 1H), 5.16 (m, 1H), 4.06 (d, J = 5.2, 2H), 2.56 (dd, J = 14.7, 6.9 Hz, 1H), 2.52 (dd, J = 14.7, 5.3 Hz, 1H), 1.67–1.55 (m, 2H), 1.34–1.21 (m, 22H), 0.88 (t, J = 6.9 Hz, 3H).

^13^C NMR (150 MHz, CDCl_3_): δ = 173.9, 172.1, 170.6, 71.1, 41.42, 41.38, 34.1, 31.9, 29.7, 29.69, 29.66, 29.56, 29.5, 29.4, 29.3, 29.2, 25.2, 22.7, 14.1.

HRMS (ESI): *m*/*z* [M+Na]^+^ calcd for C_34_H_34_D_31_NNaO_5_: 621.6701; found: 621.6681 (in positive ion mode). *m*/*z* [M-H]^−^ calcd for C_34_H_33_D_31_NO_5_: 597.6736; found: 597.6714 (in negative ion mode).

## 4. Conclusions

In conclusion, a facile procedure for the synthesis of normal or deuterated *N*-(3-acyloxyacyl)glycines **14** was developed. This strategy involved a total of seven steps from the commercially available Meldrum’s acid **4** to obtain highly pure products **14a**, **14c** and **14d**. Many reactions of this synthetic sequence led to the corresponding products with high yields, and therefore only a limited number of column chromatographic purifications were needed (three for seven steps); furthermore, none of the reactions was very water- or air-sensitive, thus they did not require anhydrous reagents/solvents/glassware or inert atmosphere. The products **14** will be used as analytical internal standards for the identification/quantification of these molecules in biological matrices. Given that they contain a chiral center, enantioseparation studies will be carried out in order to separate the two enantiomers and evaluate their individual biological activities (in addition to the biological tests performed on the racemic products).

## Data Availability

Data is contained within the article or Supplementary Materials.

## Supplementary Materials

The following supporting information can be downloaded at: https://www.mdpi.com/article/10.3390/molecules29153703/s1, ^1^H and ^13^C NMR spectra of products **13a**, **13b**, **16**, **19**, **20**, **21**, **14a**, **14b**, **14c** and **14d**.
