# Supplementary material for: Synthesis of N-(3-Acyloxyacyl)glycines, Small Molecules with Potential Role in Gut Microbiome-Endocannabinoidome Communication
Source: Molecules. 2024 Aug 5;29(15):3703. doi: 10.3390/molecules29153703 (PMC11313728; doi:10.3390/molecules29153703)
Supplement: Supplementary file 1 [file molecules-29-03703-s001.zip › molecules-3132059-supplementary.pdf]

Supporting Information for

Synthesis of *N*-(3-acyloxyacyl)glycines, small molecules with potential role in gut microbiome-endocannabinoidome communication

Rosaria Villano*[a] and Vincenzo Di Marzo[a,b]

[a] *Istituto di Chimica Biomolecolare, CNR, Via Campi Flegrei 34, 80078 Pozzuoli, Napoli, Italy*

[b] *Canada Excellence Research Chair on the Gut Microbiome-Endocannabinoidome Axis in Metabolic Health, Faculty of Medicine and Faculty of Agricultural and Food Sciences, Centre de Recherche de l'Institut de Cardiologie et Pneumologie de l'Université et Institut sur la Nutrition et les Aliments Fonctionnels, Centre NUTRISS, Université Laval, QC G1V 4G5 Quebec City, Canada*

*Corresponding author: rosaria.villano@icb.cnr.it (R. Villano)

^{1}H NMR and ^{13}C NMR spectra of the products

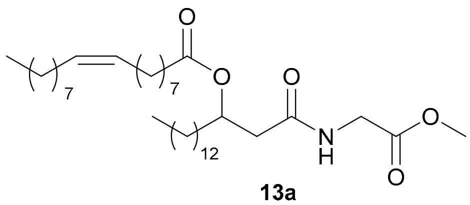

13a

^{1}H-NMR (600 MHz, $CDCl_3$)

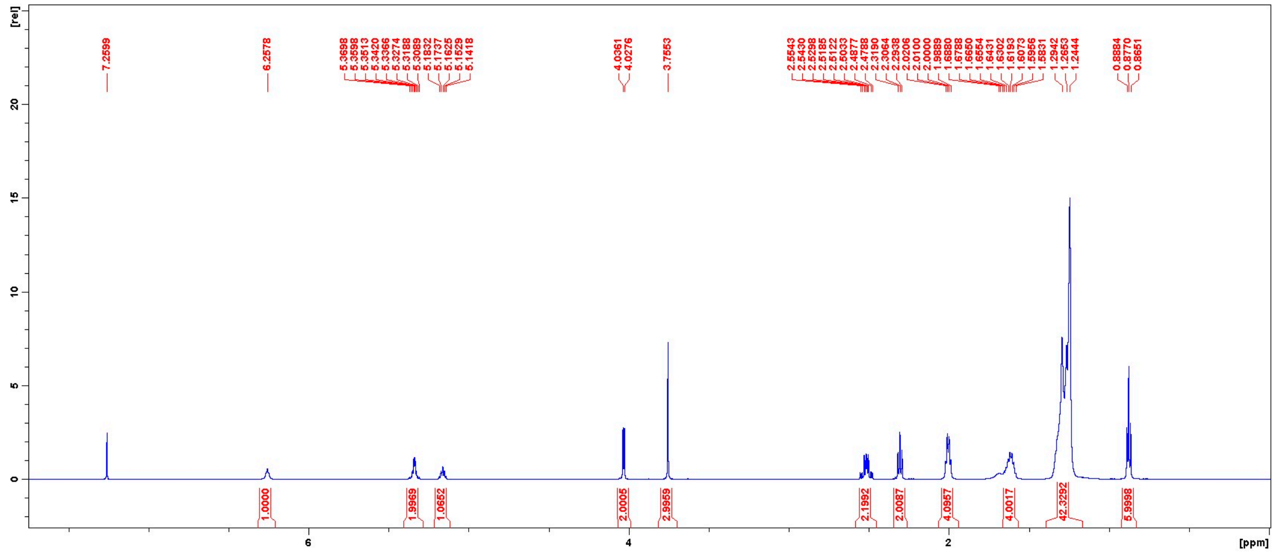

^{13}C-NMR (150 MHz, $CDCl_3$)

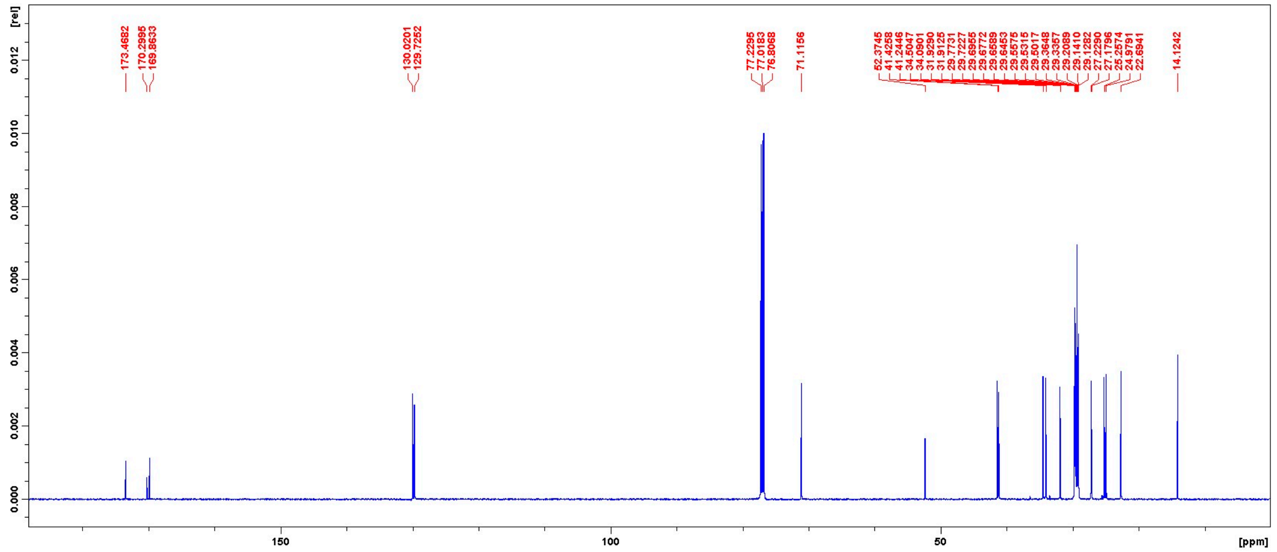

Figure S1. ^{1}H- and ^{13}C-NMR spectra ($CDCl_3$) of **13a**

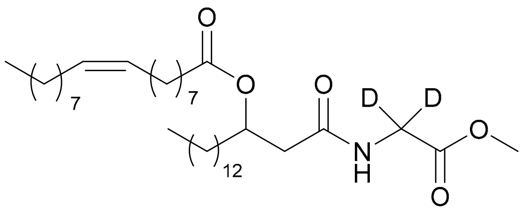

13b

^{1}H-NMR (600 MHz, $CDCl_3$)

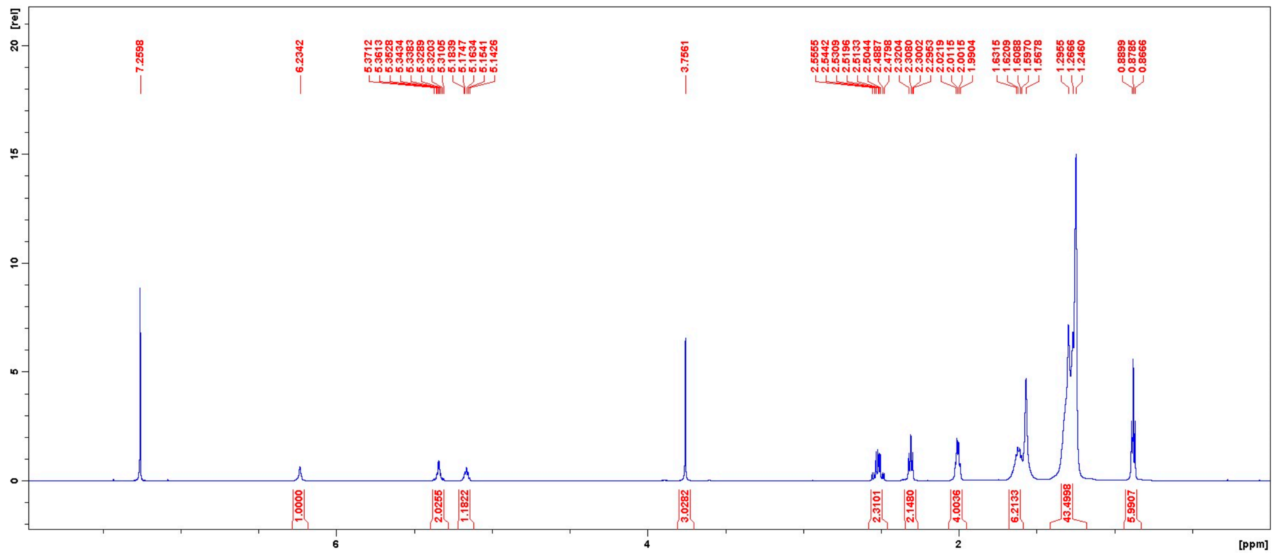

^{13}C-NMR (150 MHz, $CDCl_3$)

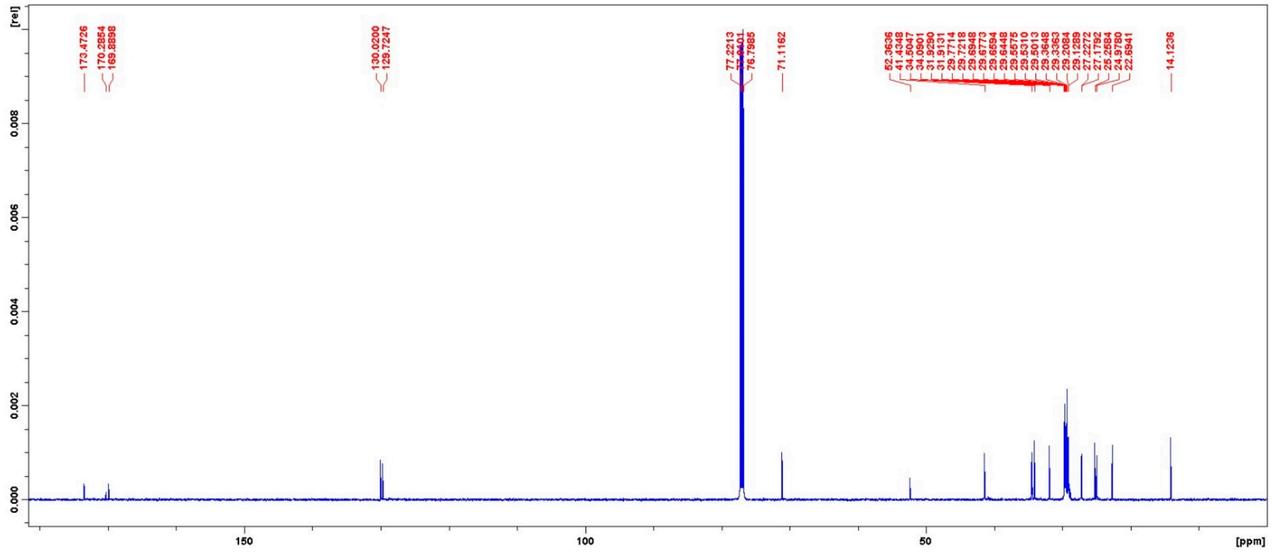

Figure S2. ^{1}H- and ^{13}C-NMR spectra ($CDCl_3$) of **13b**

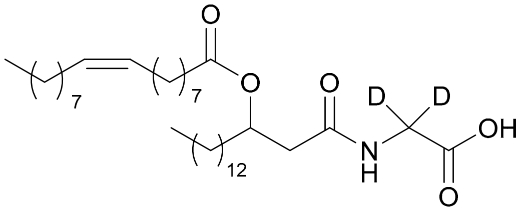

14b

^{1}H-NMR (600 MHz, $CDCl_3$)

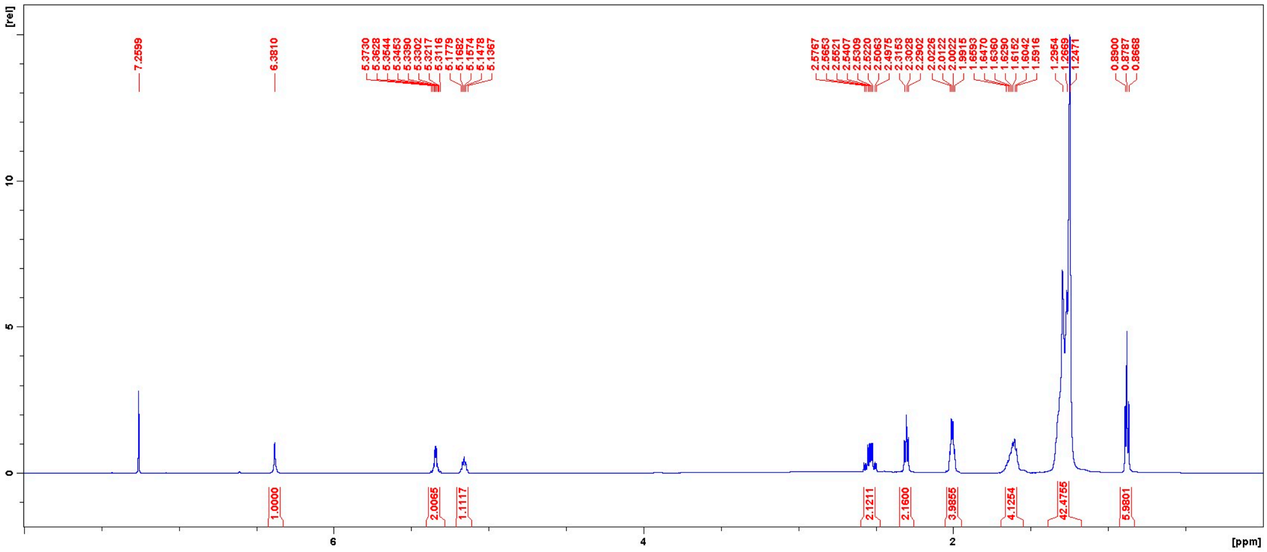

^{13}C-NMR (150 MHz, $CDCl_3$)

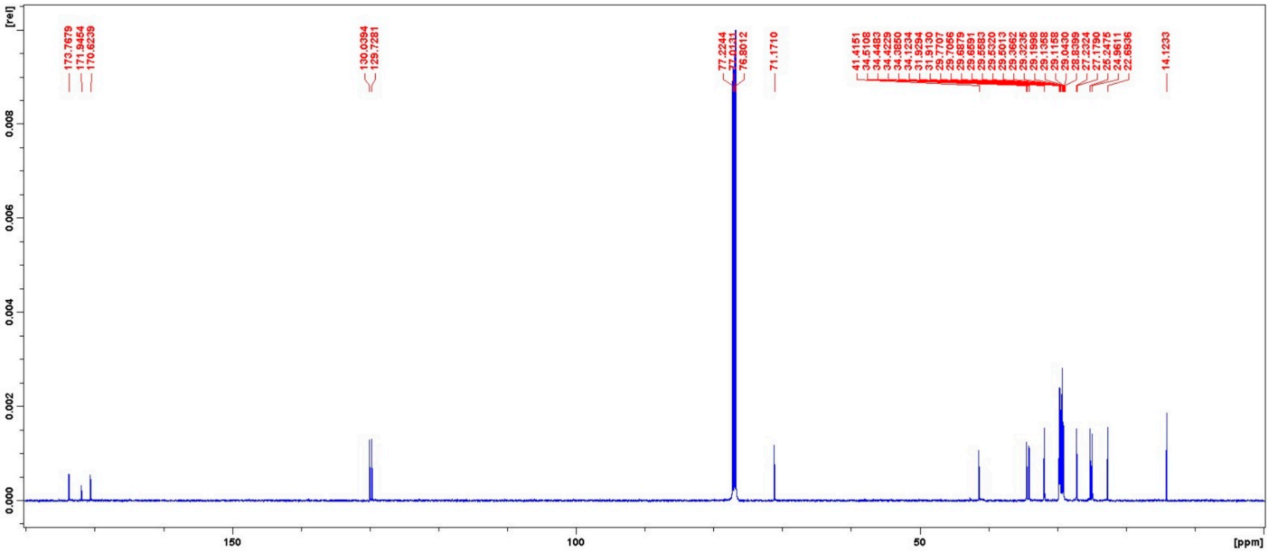

Figure S3. ^{1}H- and ^{13}C-NMR spectra ($CDCl_3$) of **14b**

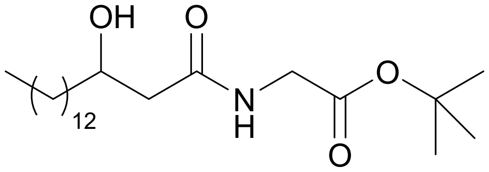

16

^{1}H-NMR (600 MHz, CDCl3)

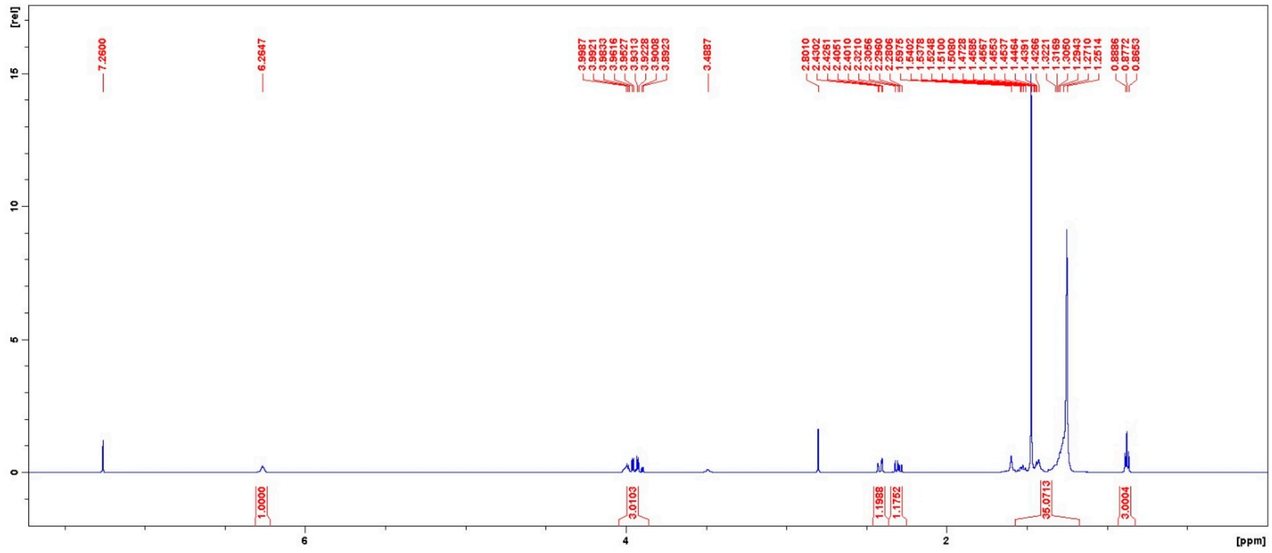

^{13}C-NMR (150 MHz, CDCl3)

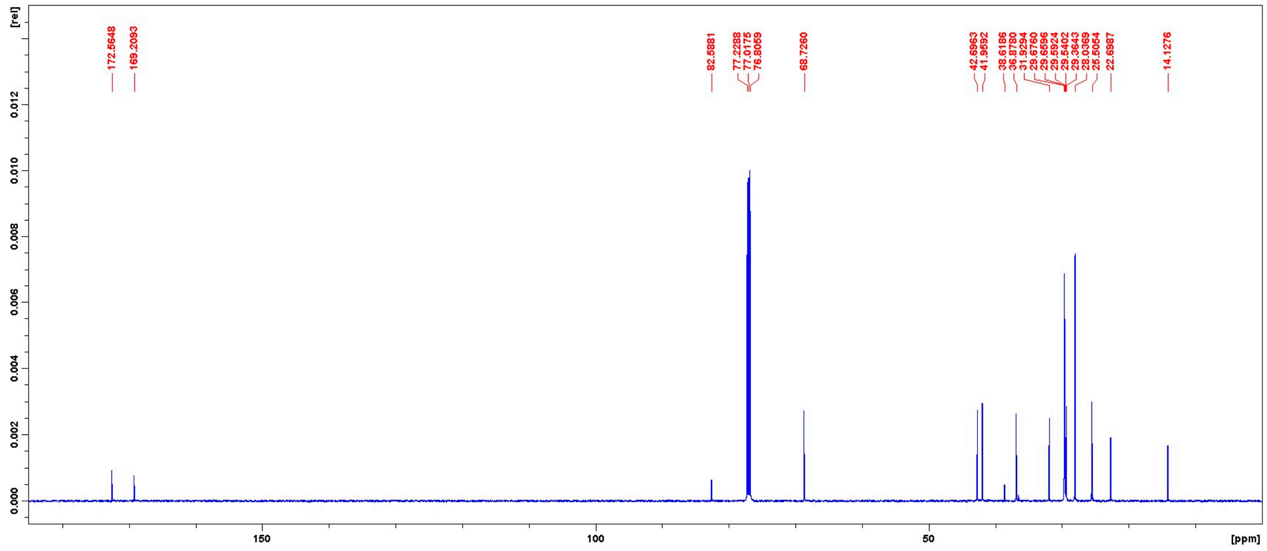

Figure S4. ^{1}H- and ^{13}C-NMR spectra (CDCl3) of **16**

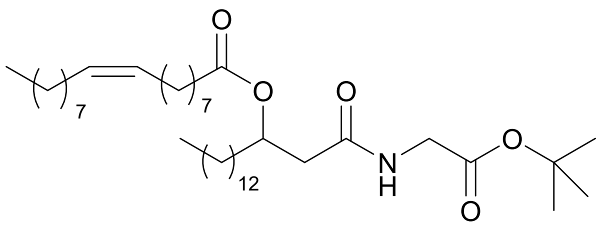

19

^{1}H-NMR (600 MHz, $CDCl_3$)

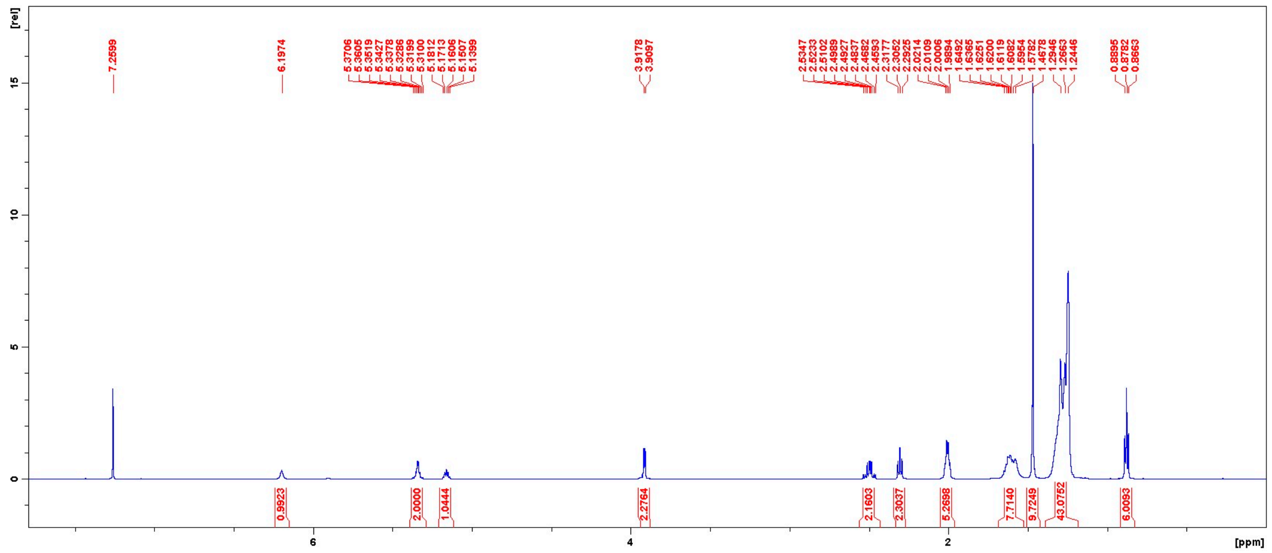

^{13}C-NMR (150 MHz, $CDCl_3$)

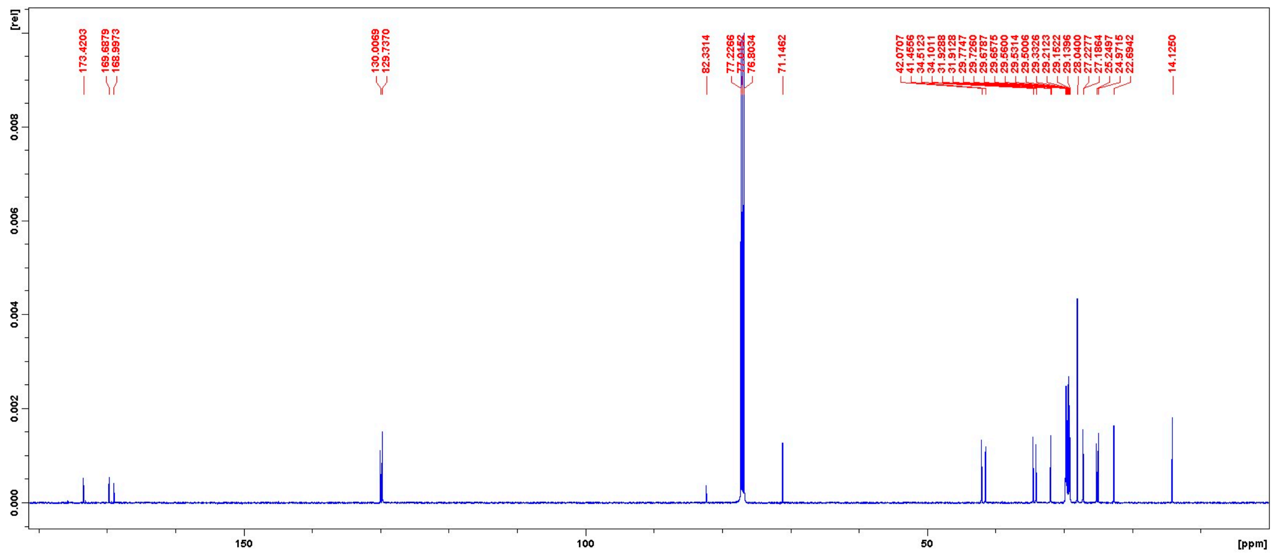

Figure S5. ^{1}H- and ^{13}C-NMR spectra ($CDCl_3$) of **19**

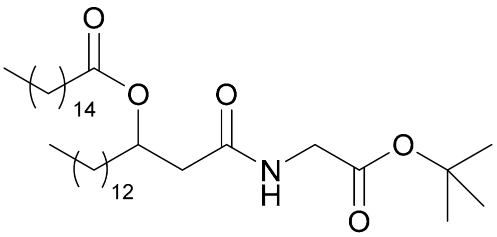

20

^{1}H-NMR (600 MHz, $CDCl_3$)

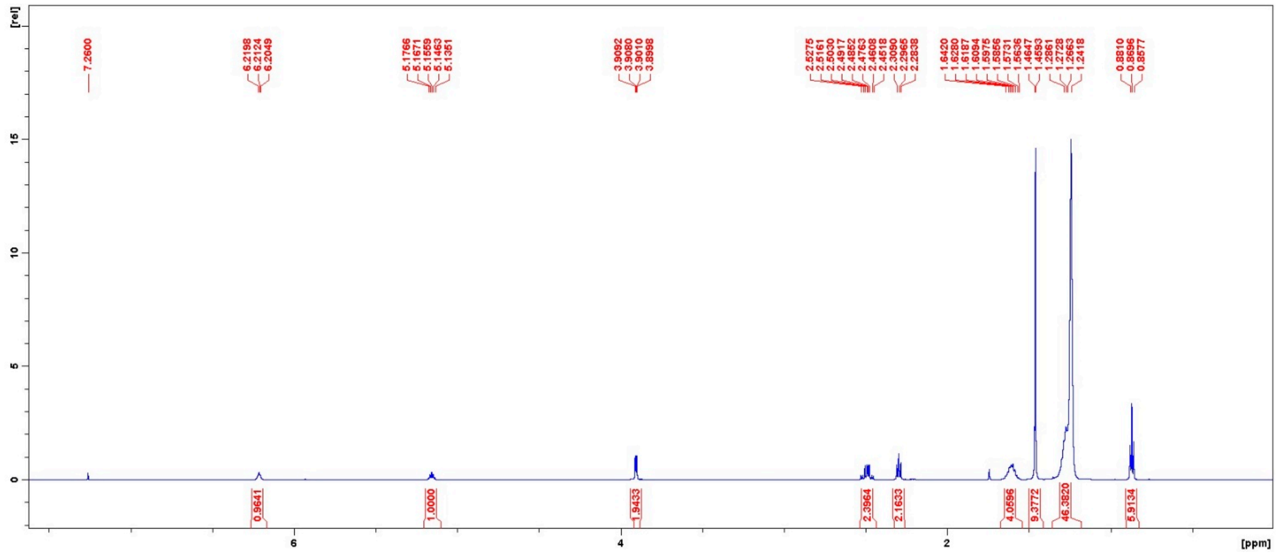

^{13}C-NMR (150 MHz, $CDCl_3$)

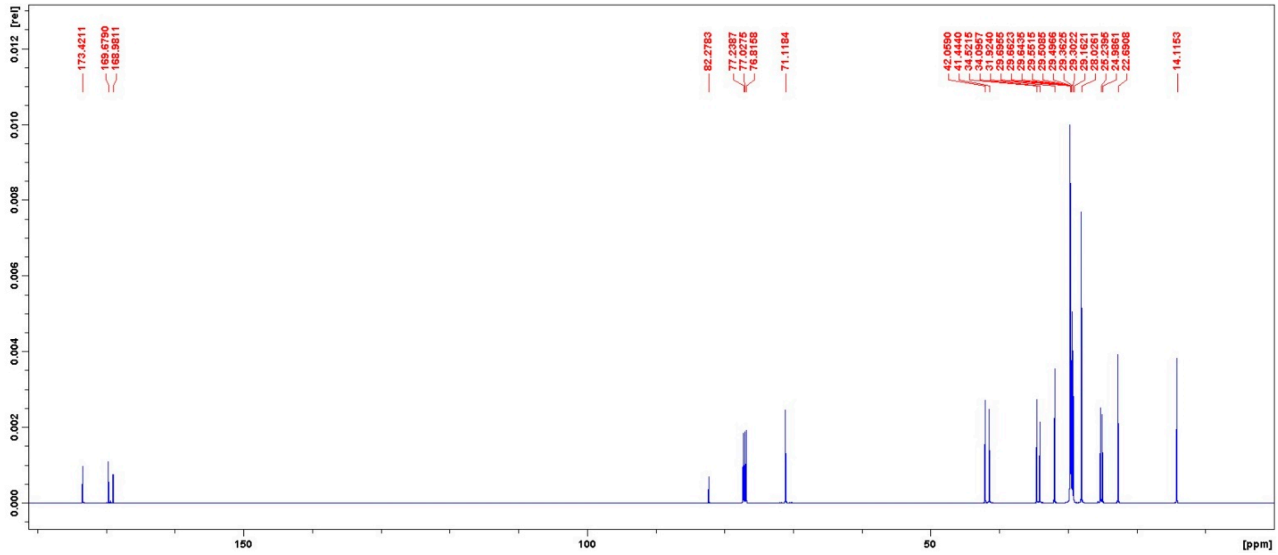

Figure S6. ^{1}H- and ^{13}C-NMR spectra ($CDCl_3$) of **20**

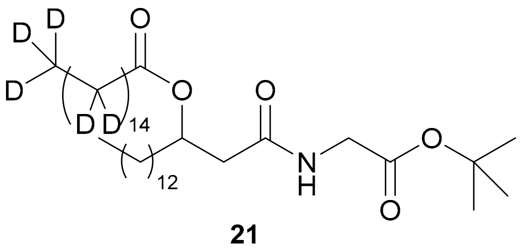

21

^{1}H-NMR (600 MHz, $CDCl_3$)

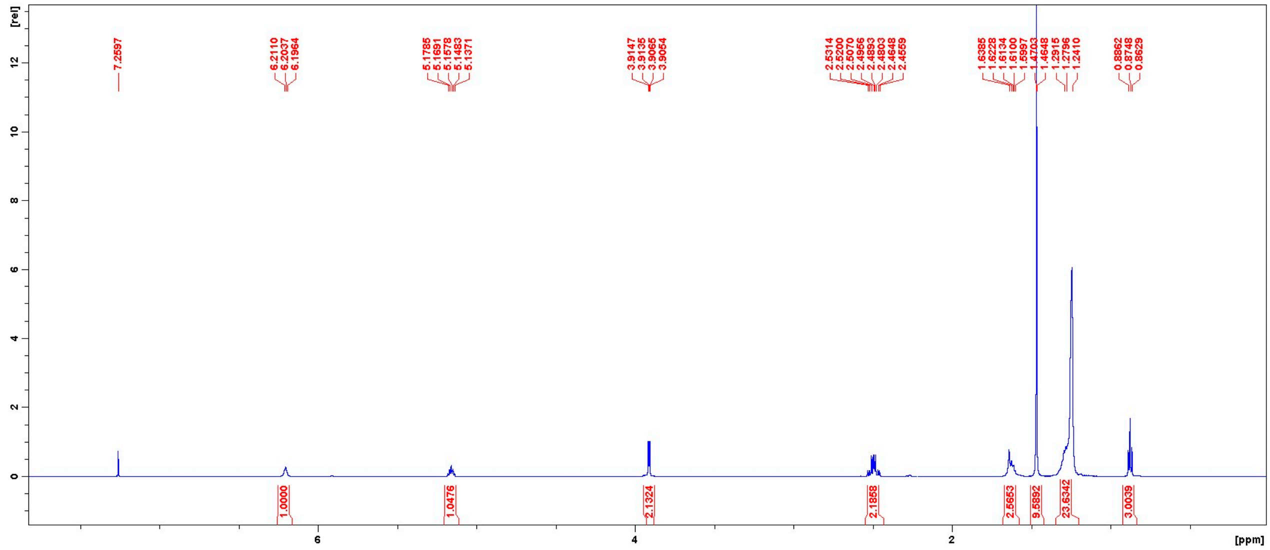

^{13}C-NMR (150 MHz, $CDCl_3$)

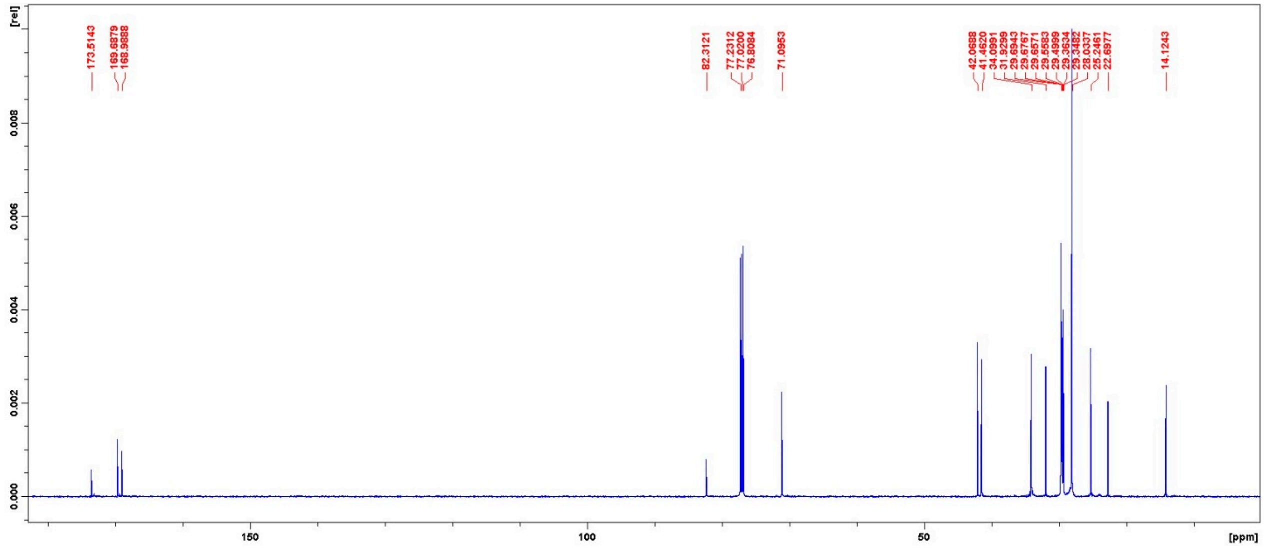

Figure S7. ^{1}H- and ^{13}C-NMR spectra ($CDCl_3$) of **21**

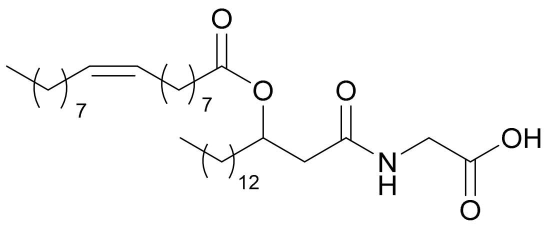

14a

^{1}H-NMR (600 MHz, $CDCl_3$)

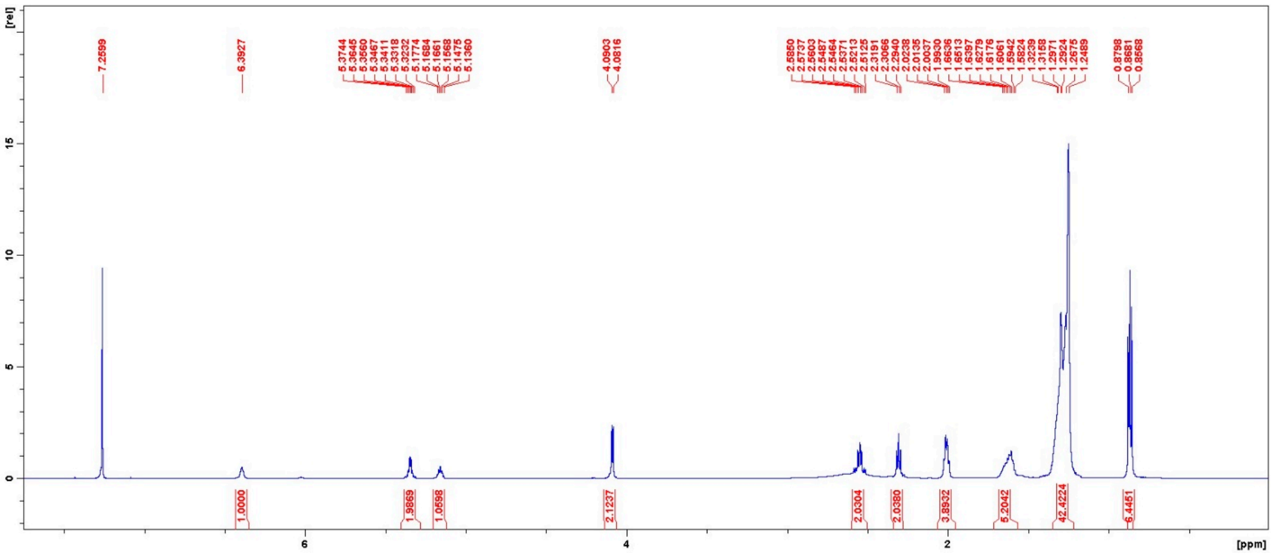

^{13}C-NMR (150 MHz, $CDCl_3$)

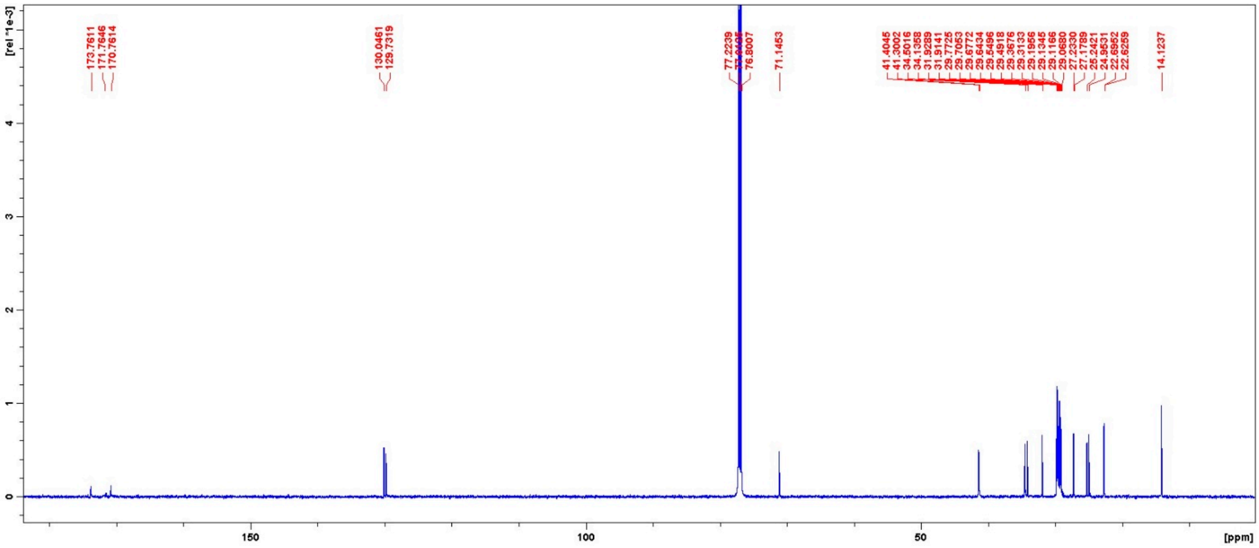

Figure S8. ^{1}H- and ^{13}C-NMR spectra ($CDCl_3$) of **14a**

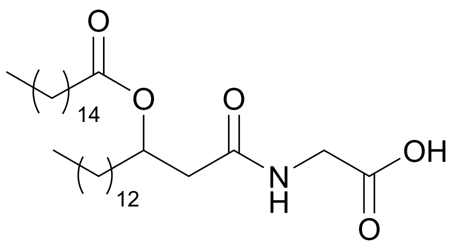

14c

^{1}H-NMR (600 MHz, $CDCl_3$)

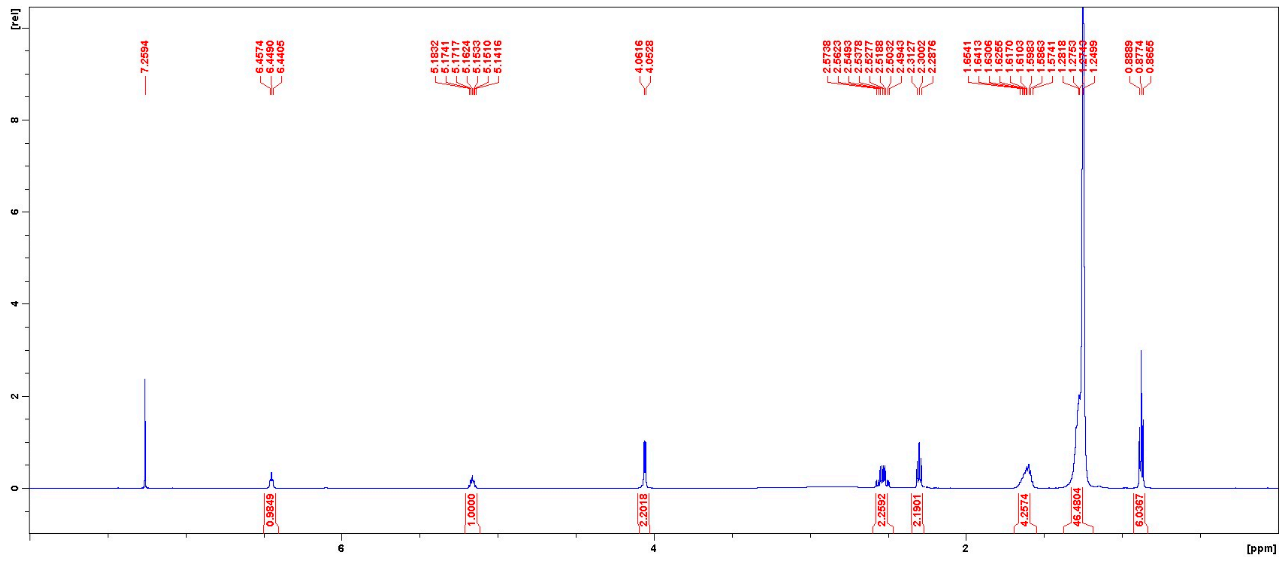

^{13}C-NMR (150 MHz, $CDCl_3$)

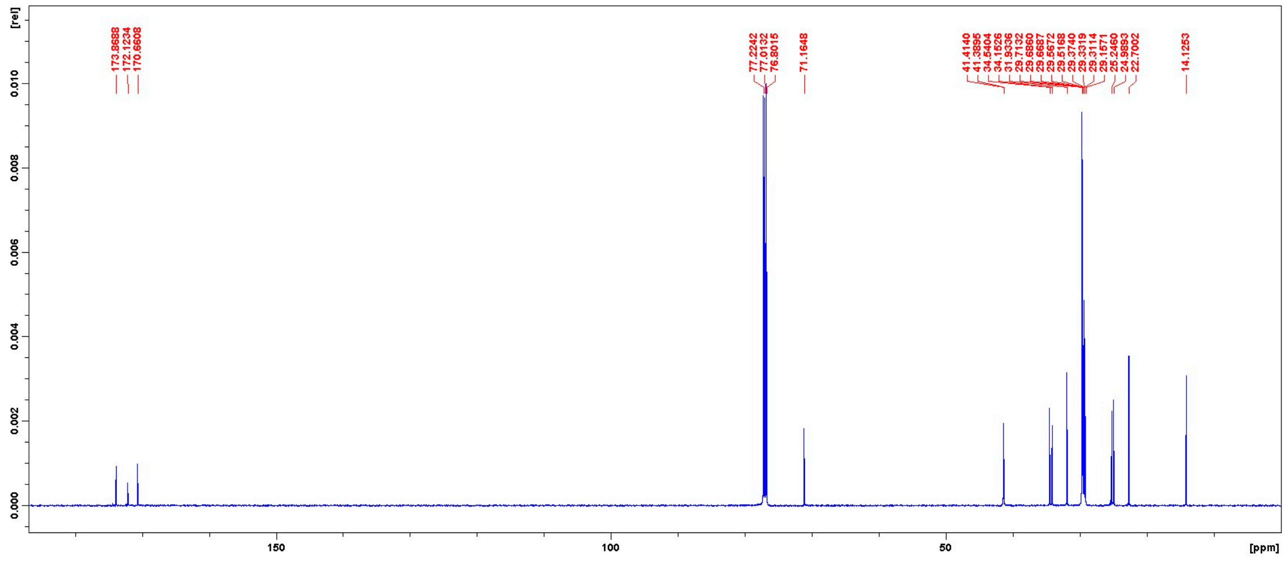

Figure S9. ^{1}H- and ^{13}C-NMR spectra ($CDCl_3$) of **14c**

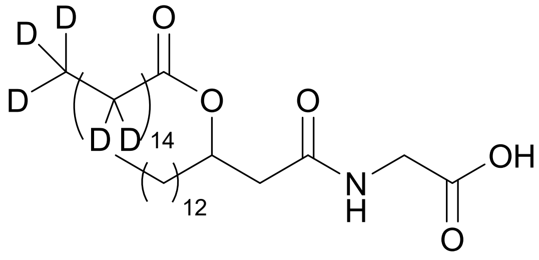

14d

^{1}H-NMR (600 MHz, $CDCl_3$)

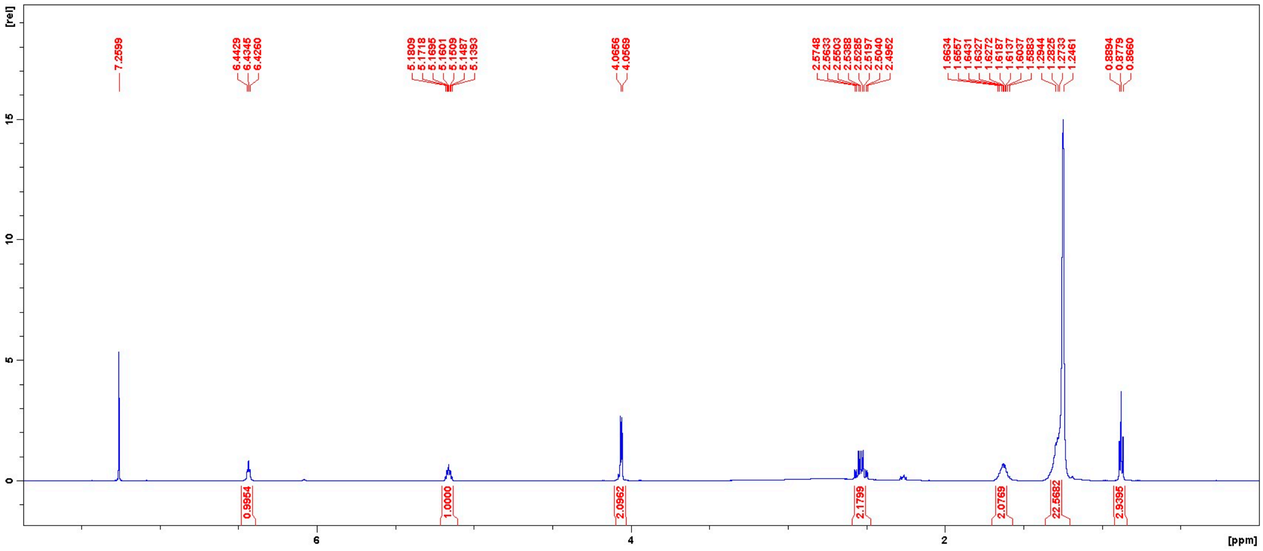

^{13}C-NMR (150 MHz, $CDCl_3$)

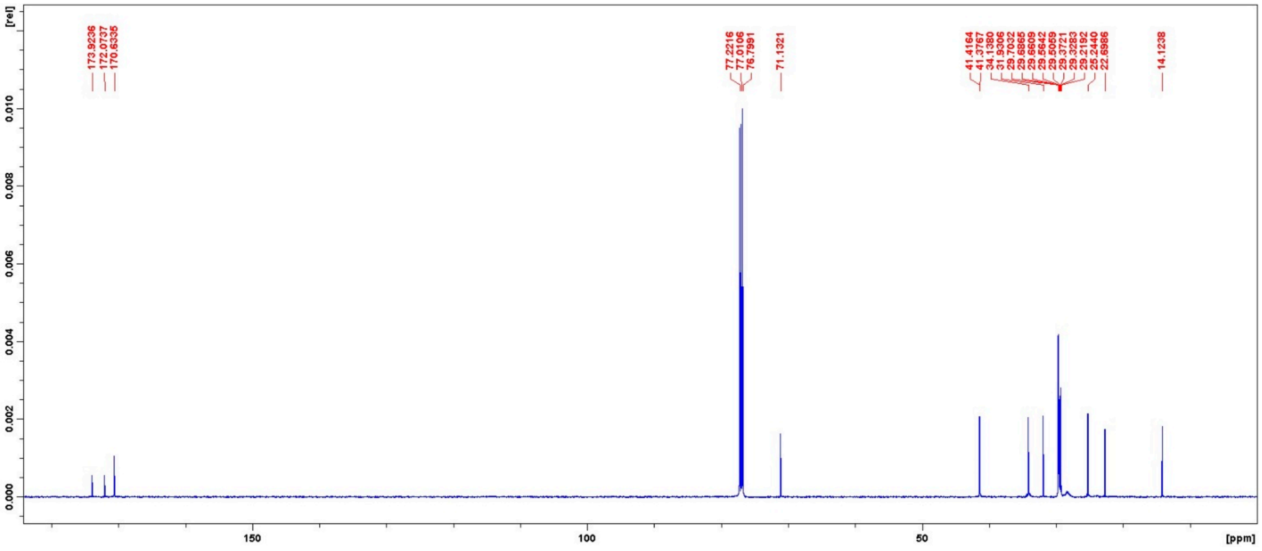

Figure S10. ^{1}H- and ^{13}C-NMR spectra ($CDCl_3$) of **14d**

Syntheses of the intermediates 6, 7, 8, 10a and 10b, carried out in accordance with the procedures reported in the literature [1]

Methyl 3-oxohexadecanoate (6): A solution of Meldrum's acid **4** (1 eq, 1.0 mmol) and pyridine (2 eq, 2.0 mmol) in THF (0.7 mL) was cooled at 0°C and myristoyl chloride (1.2 eq, 1.2 mmol) was added. The reaction mixture, warmed to rt, was stirred for 16 h. After acidification with 1N HCl, the phases were separated and the H_2O phase was extracted with EtOAc. The organic layer was dried, filtered and the solvent was removed by using a rotavapor. The crude product was dissolved in MeOH (5 mL) and the solution was refluxed for 3 h. After cooling to room temperature, the solvent was removed by using a rotavapor to give the crude β-ketoester **6**.

Methyl 3-hydroxyhexadecanoate (7): To a solution of the crude β-ketoester **6** in MeOH (1 mL) at 0°C, $NaBH_4$ (1 eq, 1.0 mmol) was added; the reaction mixture was stirred at the same temperature for 30 min and then, after neutralization with 1N HCl, it was warmed to rt. H_2O (2 mL) and EtOAc (2 mL) were added to the reaction mixture, the phases were separated and the H_2O phase was extracted with EtOAc. The combined organic layers were dried, filtered and the solvent was removed by using a rotavapor. The crude product was purified by silica gel column chromatography (eluent: light petroleum ether/EtOAc 6/1) to give the product **7** with a total yield of 90% from **4**. The spectroscopic data of **7** matched the ones reported in the literature [1]. ^{1}H-NMR (600 MHz, $CDCl_3$) δ 4.00 (m, 1H), 3.71 (s, 3H), 2.51 (dd, J = 3 Hz, 16.4 Hz, 1H), 2.40 (dd, J = 9.1 Hz, 16.4 Hz, 1H), 1.52-1.27 (m, 24H), 0.87 (t, J = 6.8 Hz, 3H).

3-Hydroxyhexadecanoic acid (8): A solution of **7** (0.25 mmol) in THF (0.1 mL) was prepared and 1N NaOH (10 eq, 2.5 mmol) was added at 0°C. The reaction mixture was stirred at 0°C for 30 min and then at rt for 2 h. After acidification with 1N HCl, the phases were separated and the H_2O phase was extracted with EtOAc. The organic layer was dried over Na_2SO_4, filtered and concentrated by using a rotavapor to afford the β-hydroxy-acid **8**. The spectroscopic data of **8** matched the ones reported in the literature [1]. ^{1}H-NMR (600 MHz, $CDCl_3$) δ 4.02 (m, 1H), 2.58 (dd, J = 3.0, 16.6 Hz, 1H), 2.47 (dd, J = 9.0, 16.6 Hz, 1H), 1.57-1.25 (m, 24H), 0.88 (t, J = 6.8 Hz, 3H).

Methyl (3-hydroxyhexadecanoyl)glycinate (10a): A solution of the β-hydroxy-acid **8** (0.22 mmol) in 2 mL EtOAc was prepared. 3Å MS, Et_3N (3 eq, 0.66 mmol), TBTU (1 eq, 0.22 mmol) and, after 1h, glycine methyl ester (2.5 eq, 0.55 mmol) were added and the reaction mixture was stirred for 16 h at rt. The reaction was quenched by adding 3 mL of H_2O and the H_2O phase was extracted with EtOAc. The organic layer was dried and concentrated by using a rotavapor. The residue was purified by column chromatography (eluent: light petroleum ether/ethyl acetate 7/3) to give **10a** (72% yield). The spectroscopic data of **10a** matched the ones reported in the literature [1]. ^{1}H-NMR (600 MHz, $CDCl_3$) δ 6.50 (bs, NH, 1H), 4.07 (dd, J = 5.4, 13.1 Hz, 1H), 4.03 (dd, J = 5.3, 13.1 Hz, 1H), 4.02 (m, 1H), 3.78 (s, 3H), 2.84 (bs, OH, 1H), 2.46 (dd, J = 2.5, 15.1 Hz, 1H), 2.35 (dd, J = 9.1, 15.1 Hz, 1H), 1.58-1.27 (m, 24H), 0.90 (t, J = 6.8 Hz, 3H).

Methyl (3-hydroxyhexadecanoyl)glycinate-d_2 (10b): According to the synthesis of **10a**, **10b** was prepared by using deuterated glycine methyl ester. The spectroscopic data of **10b** matched the ones reported in the literature [1]. ^{1}H-NMR (600MHz, $CDCl_3$) δ 6.43 (bs, NH, 1H), 4.02 (m, 1H), 3.78 (s, 3H), 2.84 (bs, OH, 1H), 2.46 (dd, J = 2.5, 15.1 Hz, 1H), 2.34 (dd, J = 9.1, 15.1 Hz, 1H), 1.58-1.26 (m, 24H), 0.90 (t, J = 6.8 Hz, 3H).

References

1. Villano, R.; Tinto, F.; Di Marzo, V. Facile and Sustainable Synthesis of Commendamide and its Analogues. *Front. Chem.* **2022**, *10*, 858854. DOI: 10.3389/fchem.2022.858854
